# Recurrent Bleeding From Cervical Varices in Pregnancy: A Case Report and Literature Review

**DOI:** 10.7759/cureus.96050

**Published:** 2025-11-03

**Authors:** Tomoki Nobumasa, Masahito Takakura, Hikaru Imatake, Asako Inohaya, Maya Komatsu, Ayaka Yamaguchi, Yoshitsugu Chigusa, Haruta Mogami, Masaki Mandai

**Affiliations:** 1 Department of Gynecology and Obstetrics, Japanese Red Cross Otsu Hospital, Otsu, JPN; 2 Department of Gynecology and Obstetrics, Kyoto University Hospital, Kyoto, JPN; 3 Department of Obstetrics and Gynecology, Kobe City Medical Center General Hospital, Kobe, JPN

**Keywords:** abnormal placentation, cervical varices in pregnancy, cesarean section, gauze compression, massive bleeding

## Abstract

Cervical varices are a rare pregnancy complication that can result in recurrent or massive genital bleeding, posing a risk for preterm delivery. There is no established management strategy, and individualized treatment is required. A 32-year-old woman, gravida 5 para 0, with a bicornuate uterus and multiple uterine fibroids, was referred at 21 weeks and three days of gestation due to persistent vaginal bleeding. She was diagnosed with cervical varices associated with a low-lying anterior placenta. During a 15-week inpatient course, she experienced approximately 20 episodes of bleeding, some exceeding 1000 mL and requiring hemodynamic support. Each episode was successfully managed by direct gauze compression. A total of 30 units of red blood cells (RBCs) and six units of fresh frozen plasma (FFP) were transfused. At 36 weeks and three days of gestation, cesarean delivery was performed under spinal anesthesia, and a healthy male infant was delivered. The cervical varices regressed postoperatively and had resolved completely by one month postpartum. The conclusion is that gauze compression is an effective first-line approach for managing cervical variceal bleeding and can help prolong gestation in affected pregnancies.

## Introduction

Cervical varices are a rare complication of pregnancy and can pose a risk of preterm birth due to massive genital bleeding during the course of gestation [1-4]. Recurrent bleeding may also occur during pregnancy, and effective control of hemorrhage is crucial; however, no standardized management strategy has been established. Here, we report a case of pregnancy complicated by cervical varices with recurrent genital bleeding, in which pregnancy was successfully prolonged until 36 weeks gestation through repeated hemostatic compression and blood transfusions.

## Case presentation

**Figure 1:**
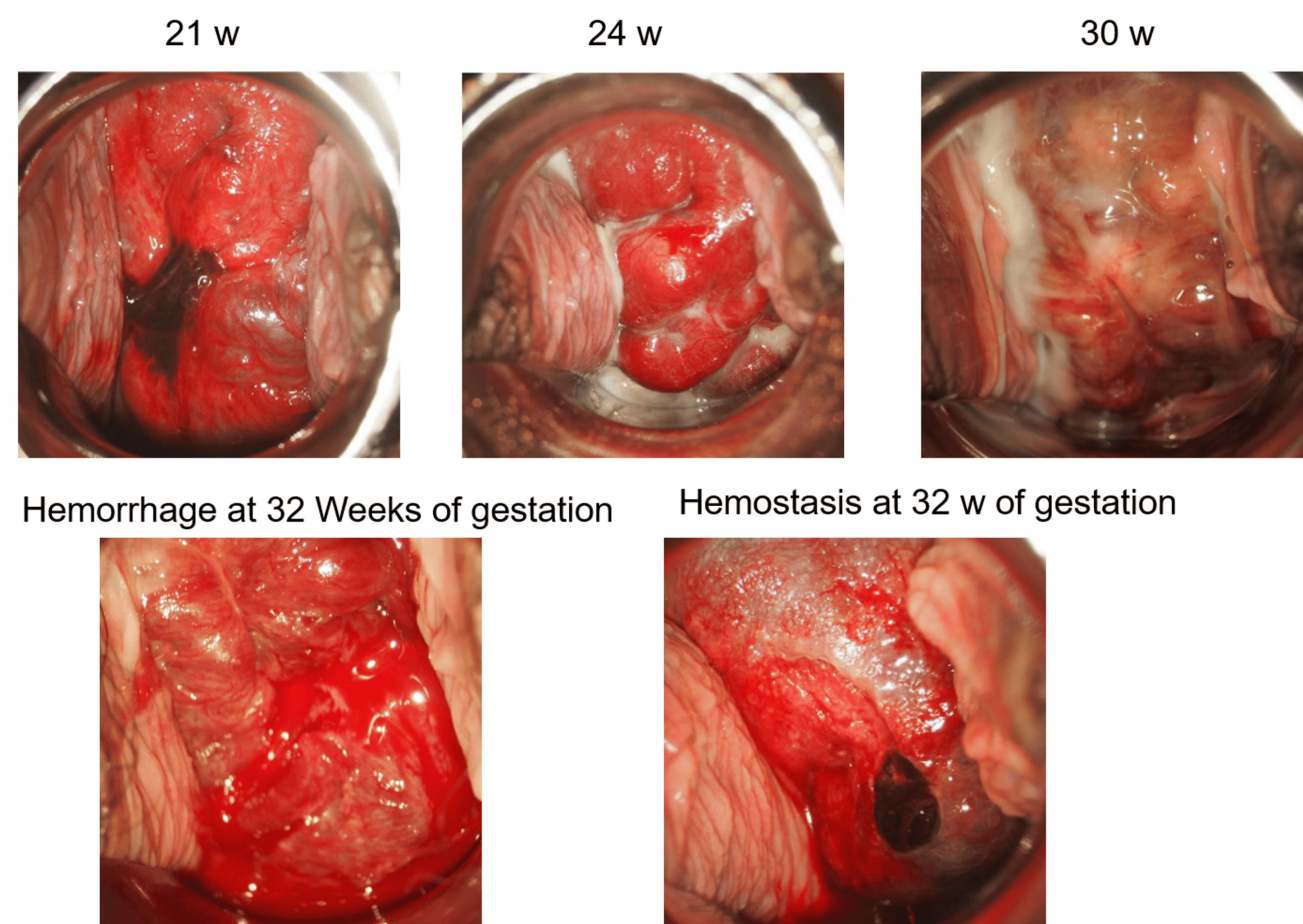
Macroscopic features of cervical varices at 21, 24, 30, and 32 weeks of gestation. Upper panels: serial changes of the cervical varices. Lower panels: hemorrhage from cervical varices (left) and hemostasis after gauze compression (right) at 32 weeks of gestation. Note that engorged vessels replace the cervix at 21 and 24 weeks of gestation (upper panel, left and middle). Varices temporarily regressed at 30 weeks of gestation (upper panel, right).  Hemostasis is accomplished by gauze compression after massive bleeding at 32 weeks of gestation (lower panel, left). Cervical external os cannot be identified due to large varices during pregnancy.

**Figure 2:**
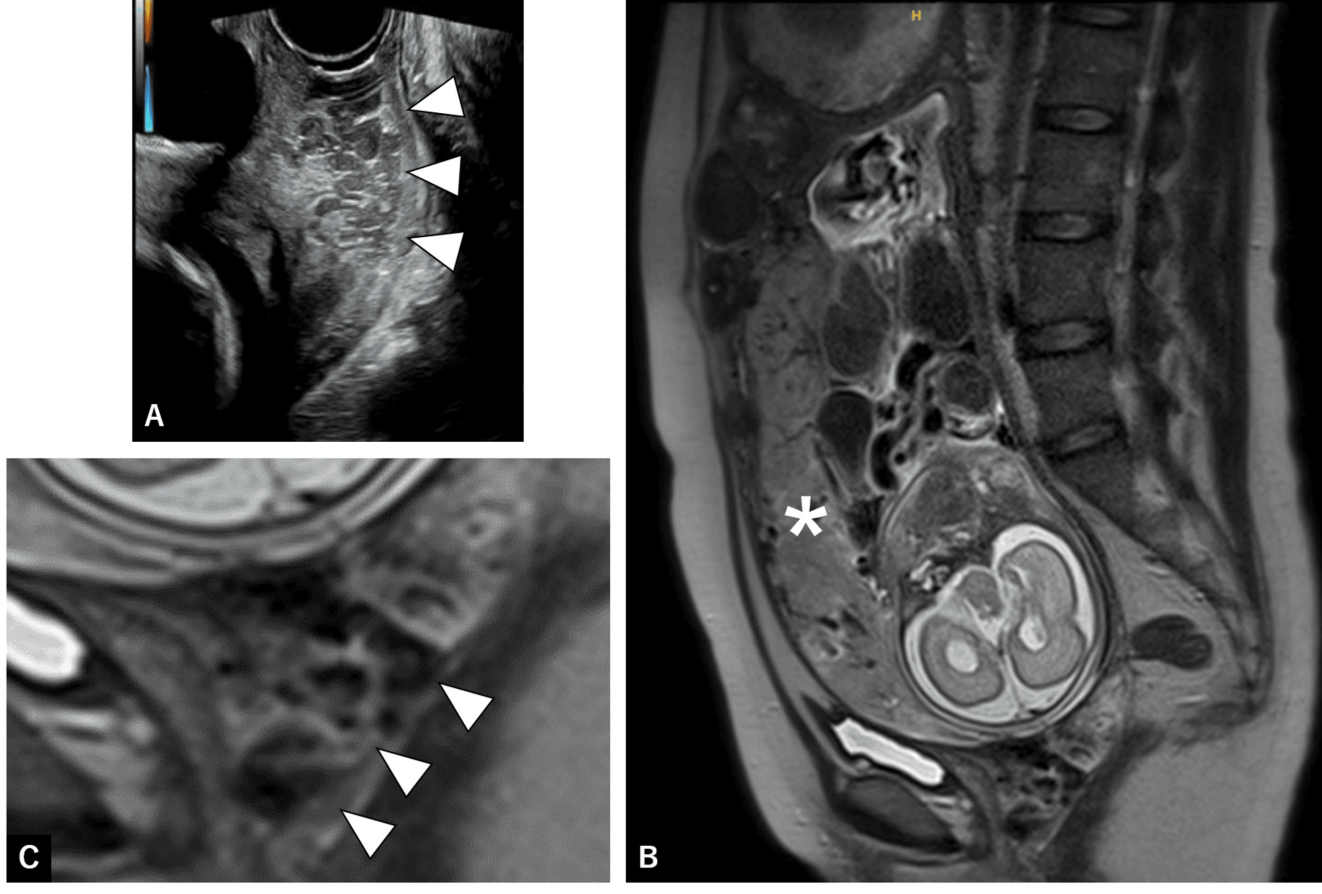
Ultrasound and MRI images (A) Transvaginal ultrasonography at 22 weeks of gestation. Arrowheads indicate dilated vessels within the cervix. (B, C) Magnetic resonance imaging (MRI) at 26 weeks of gestation. (B) T2-weighted sagittal view shows the low-lying placenta (asterisk) attached to the anterior uterine wall. (C) Enlarged image of (B). Note that dilated vessels are replacing the anterior cervical lip.

A 32-year-old pregnant woman, gravida 5 para 0, with a history of four miscarriages, was referred to our hospital at 21 weeks and three days of gestation due to persistent vaginal bleeding. During evaluation for recurrent pregnancy loss, she was diagnosed with a bicornuate uterus and multiple uterine fibroids. She conceived spontaneously. On speculum examination, the cervix was circumferentially replaced by engorged vessels (Fig. 1). There were no varices on the vaginal walls. Transvaginal ultrasonography showed prominent dilated vessels within the cervix (Fig. 2A). A diagnosis of cervical varices in pregnancy was made, and the patient was admitted for inpatient management. The laboratory tests are shown in Table 1. The placenta was located anteriorly and low-lying. Magnetic resonance imaging (MRI) at 26 weeks of gestation demonstrated dilated vessels extending from the cervix to the internal os, without continuity with the placenta (Fig. 2B, 2C).

**Table 1:** Laboratory tests at the admission WBC: white blood cell, PT: prothrombin time, APTT: activated partial thromboplastin time, CRP: C-reactive protein

Tests	Results	Normal range
WBC	9.63×10⁹/L	3.30-8.60×10⁹/L
Hemoglobin	8.7 g/dL	11.6-14.8 g/dL
Platelets	227×10⁹/L	158-348×10⁹/L
PT	14.8 sec	10.2-13.4 sec
APTT	36.5 sec	24-39 sec
Fibrinogen	793 mg/dL	200-400 mg/dL
Antithrombin	97.2 %	83-118 %
D-dimer	0.7 μg/mL	<1.0 μg/mL
CRP	3.93 mg/dL	<0.14 mg/dL

**Figure 3:**
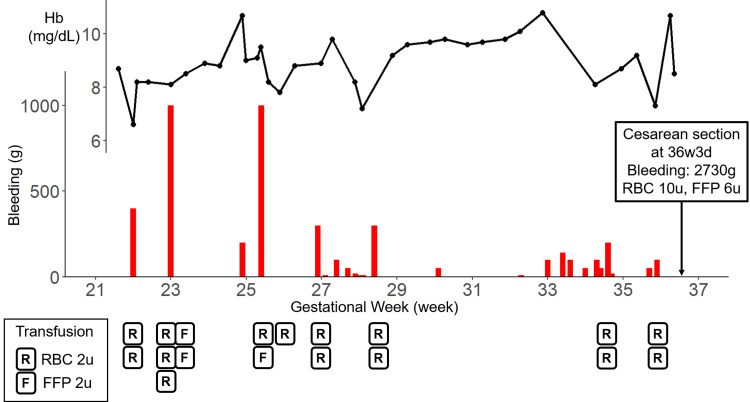
Clinical course of the patient. The upper line graph shows the changes in hemoglobin (Hb) concentration during pregnancy. The lower bar graph shows the amount of bleeding. The amount of blood transfusion (R: two units of red blood cells, and F: two units of fresh frozen plasma) in each hemorrhage event is indicated below.

During hospitalization, she experienced repeated episodes of bleeding. The frequency ranged from two to three times per week, and the amount of bleeding ranged from 10 g to over 1000 g, with some episodes resulting in hypovolemic shock with over 1000 g bleeding at 23 weeks and 25 weeks of gestation, while others involved minor but persistent bleeding. Each bleeding episode was managed with approximately five minutes of direct gauze compression, which successfully achieved hemostasis. Blood transfusions with red blood cells (RBCs) and fresh frozen plasma (FFP) were administered according to the severity of bleeding and anemia. The clinical course of treatment is summarized in Fig. 3. Serial observations of the cervical varices were possible during her hospitalization. The morphology of the varices varied over time (Fig. 1). Around 30 weeks of gestation, the varices appeared reduced in size, and bleeding episodes decreased. However, at 32 weeks, the engorged vessels became more prominent again, and bleeding recurred. From 34 weeks onward, bleeding occurred daily, which led to significant anemia requiring transfusion. Considering fetal maturity, we elected to terminate the pregnancy at 36 weeks.

The placental position did not improve during the course of pregnancy, and it remained a low-lying placenta at 36 weeks of gestation. Given a bicornuate uterus, multiple uterine fibroids, the low-lying anterior placenta, and the risk of rupture of cervical varices during vaginal delivery, an elective cesarean section was planned. Over the 15-week inpatient course, she experienced approximately 20 bleeding episodes and required transfusion of 30 units of RBCs and six units of FFP. Cesarean delivery was performed at 36 weeks and three days of gestation under spinal anesthesia with a vertical skin incision and low transverse uterine incision. A male infant weighing 3056 g was delivered with the Apgar scores of 8/9 and the umbilical artery pH of 7.339.

The placenta was spontaneously separated, but after placental removal, active bleeding was noted from the area of the internal os. It was controllable with some sutures and gauze compression. To prevent further hemorrhage due to rupture of cervical varices, a double-balloon intrauterine catheter was placed, compressing the cervix from both the uterine and vaginal sides. The estimated blood loss during the operation was 2730 mL, and the patient received 10 units of RBCs and six units of FFP.

**Figure 4:**
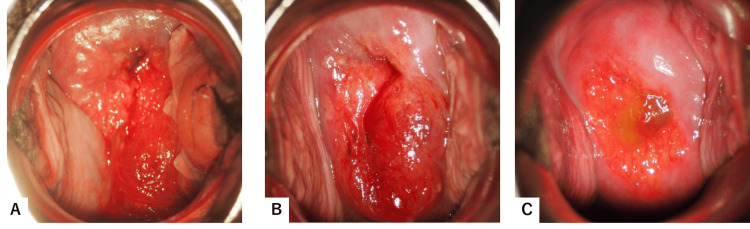
Macroscopic features of the cervices after parturition. Postoperative day one (A), day four (B), and one month (C). Note that cervical external os cannot be identified until one month of delivery due to complete shrinkage of varices.

On the day after surgery, the double-balloon intrauterine catheter was removed vaginally, and by speculum examination, the cervical varices appeared deflated, with no active bleeding from the uterus (Fig. 4A). By postoperative day four, the varices had further regressed, and the anterior lip and external os became identifiable (Fig. 4B). The patient was discharged without complications. At the one-month postpartum visit, the cervical varices had completely resolved, and the cervix had returned to a normal appearance (Fig. 4C).

## Discussion

While varices of the lower extremities and vaginal wall are common during pregnancy, cervical varices are rare. Historically, exposure to diethylstilbestrol (DES) was considered a possible cause of cervical varices, but recent reports have instead shown a strong association with abnormal placental positioning, such as placenta previa or low-lying placenta. A review of cases found via PubMed revealed that 66.7% (14 of 21 cases) were associated with abnormal placental location (Table 2) [1-21]. In our case, the patient had a low-lying anterior placenta, in addition to multiple uterine fibroids and a bicornuate uterus. There are no prior reports linking uterine anomalies or multiple fibroids directly to cervical varices, so the association of cervical varices and uterine anomalies or fibroids is unclear.

**Table 2:** Published cases of the cervical varices in pregnancy

No	Study (first author, year)	Abnormal placentation	Antepartum bleeding	Treatment	GA at termination	Mode of delivery	Blood loss	Transfusion at delivery	Postpartum bleeding
1	T. Hurton, 1998 [1]	-	Several times	Conservative, RBC 2u	33 w	emCS	5000 ml	RBC 5u	Yes, hysterectomy
2	K. Yoshimura, 2004 [2]	Low-lying	16 w	Conservative	27 w	emCS	920 ml	No	No
3	JP.Kusanovic, 2006 [3]	Previa until 29 w	Several times	Conservative, RBC 4u	32 w	emCS	N/A	RBC 2u	Yes, packing
4	Y. Kumazawa, 2007 [4]	Previa	32 w	Conservative	32 w	emCS	1814 ml	Autologous	No
5	RN. Sammour, 2011 [5]	Low-lying	No	No	37 w	CS	1000 ml	No	No
6	YE. Sukur, 2011 [6]	Previa	No	No	38 w	emCS	1500 ml	No	No
7	JV. Brown, 2012 [7]	N/A	15 w	Suture ligation, Gelfoam (15 w)	N/A	NA	1100 ml	No	N/A
8	Y. Kurihara, 2013 [8]	Low-lying	No	No	37 w	CS	3610 ml	RBC 4u	No
9	B. O'Brien, 2013 [9]	Previa	19 w, 33 w	Conservative	34 w	CS	500 ml	No	No
10	J. Lesko, 2014 [10]	Previa	16 w	UAE, D&E, RBC 2u, FFP 2u	17 w	UAE, D&E	N/A	No	No
11	K. Chyjek, 2015 [11] (Case #1)	Low-lying until 26 w	15 w	Cerclage (15 w)	38 w	CS	1000 ml	No	No
12	(Case #2)	Low-lying	2 times	Conservative	38 w	CS	1000 ml	No	No
13	M. Tanaka, 2016 [12]	Previa	33 w	Conservative	36 w	CS	3200 ml	RBC 3u	No
15	MY. Peng, 2018 [13]	-	31 w	Suture ligation (31 w)	33 w	CS	850 ml	No	No
14	JR. Wax, 2018 [14]	Low-lying until 19 w	12 w	Conservative	33 w	CS	800 ml	No	No
16	JE. Park, 2019 [15]	N/A	8 w	Conservative	8 w	D&E	N/A	N/A	N/A
17	Y. Fujibe, 2021 [16]	Low-lying	20 w, 21 w, 22 w	Cerclage (22 w), transfusion	34 w	CS	3643 ml	Yes	No
18	E. Gonzalez-Bosquet, 2021 [17]	Previa	16 w, 20 w	Pessary (21 w)	36 w	emCS	N/A	N/A	No
19	N. Poliektov, 2022 [18]	Low-lying	No	Ligation, cerclage (12 w), RBC 6u, FFP 1u	37 w	CS	800 ml	No	No
20	CK. Wong, 2022 [19] (Case #1)	-	30 w	Conservative	36 w	VD	N/A	N/A	No
21	(Case #2)	-	34 w, 36 w	Conservative	36 w	emCS	800 ml	No	No
22	(Case #3)	-	4 times	Conservative	36 w	emCS	200 ml	No	No
23	(Case #4)	-	3 times	Conservative	29 w	emCS	300 ml	No	No
25	N. Saedi, 2023 [20]	Previa	14 w, 18 w	Conservative, RBC 4u	37 w	CS	2000 ml	RBC 4u, FFP 2u, PC 2u	Yes, hysterectomy
24	J. Youssef, 2023 [21]	-	35 w, 36 w	Conservative	36 w	CS	800 ml	RBC 2	No
26	Present case	Low-lying	20 times	Conservative, RBC 30u, FFP 6u	36 w	CS	2730 ml	RBC 10u, FFP 6u	No

The mechanism of cervical varices formation is presumed to be that increased blood flow to the cervix due to abnormal placental positioning exceeds the drainage capacity of the cervical veins, leading to the accumulation of blood in the form of varices [8]. In this case, serial observation of cervical varices was possible, and the morphology of the varices differed at each episode. This suggests a repeated cycle of the rupture of varices and the formation of new varices.

In terms of acute management, gauze compression appears to be the first-line method to achieve hemostasis. Although there are reports of hemostasis achieved using cervical sutures or pessaries [7,11,13,16-18], in this case, surgical suturing was not attempted due to the large size of the varices and the inability to visualize the cervix adequately. As a result, 20 episodes of bleeding with gauze compression and a large volume of blood transfusion (30 units of RBC and 6 units of FFP) were required. Among previously reported cases, 11 experienced recurrent bleeding [1,3,9,11,16,17,19-21], and 5 required blood transfusion [1,3,10,18,20] during pregnancy; the frequency of bleeding episodes and the volume of blood transfusion in this case were markedly higher. In our case, gauze was replaced every 24 hours to minimize infection risk, and prophylactic antibiotics were administered. Fortunately, no infectious complications occurred. Although multiple transfusions were required, transfusion was unavoidable in anticipation of further hemorrhagic episodes.

Cesarean delivery is typically selected in cases of cervical varices to avoid rupture of the varices during vaginal delivery. Nevertheless, even with cesarean delivery, there are three reports of massive postpartum hemorrhage caused by ruptured cervical varices [1,3,20]. At our institution, prophylactic intrauterine balloon tamponade is routinely used in cases of abnormal placental location (placenta previa and low-lying placenta) to prevent hemorrhage from the placental implantation site. In this case, we employed a double-balloon intrauterine catheter-positioning one balloon in the uterus and another in the vagina to sandwich the cervix-both to compress the placental site and to reduce the risk of variceal rupture. This approach may be effective even in cases without placental abnormalities.

## Conclusions

Cervical varices in pregnancy are rare but can cause recurrent and life-threating massive bleeding, posing a risk for preterm birth. Our case demonstrates that simple gauze compression can serve as an effective first-line measure to achieve rapid hemostasis, even after massive hemorrhage, and allow for successful prolongation of gestation. Persistent and individualized management, including repeated transfusions and carful inpatient monitoring, is essential to improve both maternal and neonatal outcomes. Furthermore, proactive preparation for delivery, including consideration of intrauterine balloon tamponade and cesarean section in the setting of abnormal placental location, may help minimize intrapartum and postpartum hemorrhagic complications.
